# Impact-echo data and crack classification of six prestressed reinforced concrete sleepers of type B70

**DOI:** 10.1016/j.dib.2026.113299

**Published:** 2026-10-01

**Authors:** Christoph Strangfeld, David Ringeloth

**Affiliations:** Bundesanstalt für Materialforschung und -prüfung (BAM), Division 8.2 “Non-Destructive Testing Methods for Civil Engineering”, Unter den Eichen 87, Berlin, 12205, Germany

**Keywords:** Air-coupled impact-echo, Crack detection, Supersonic jet flow, Non-destructive testing, Civil engineering, B70 sleeper, railway infrastructure

## Abstract

This article presents non-destructive testing data from six prestressed reinforced concrete sleepers of type B70. The investigation methods employed were visual inspection and impact-echo. Impact Echo testing was performed with three different actuator types, including air-coupled impact-echo actuation. For data acquisition, the same accelerometer was used for all actuators. Two of the six sleepers were factory-new. The other four were in service for several years and show serious deterioration and crack patterns. The crack types and corresponding damage classes are documented in the crack catalogues.

This is the first time air-coupled impact-echo data have been published. The data consist of a crack catalogue based on visual inspection, time series of the accelerometer mounted on the top surface, and high-resolution photographs of the sleepers. This dataset is unique as it enables a direct comparison of different types of actuation on slender geometries made of concrete. Air-coupled and manual impact-hammer actuation was performed on all sleepers. Actuation using a commercial device with an electromechanically mounted impact-hammer was applied only to one intact and one damaged sleeper, as the resulting spectrograms are very similar to those obtained using manual impact-hammer actuation. The dataset enables the community to verify and reevaluate the data. This should give confidence in the new approach and motivate others to develop advanced data processing to increase prediction accuracy. Furthermore, the actuation needs just pressurised air, delivered through common air gun nozzles. Thus, other research groups and inspection companies might adopt this method and use our data or processing approach for fast and efficient comparison. This article accompanies the research article “Air-coupled impact-echo actuation applied on concrete sleepers for crack detection” published in the Journal of Nondestructive Evaluation (Springer Nature). There, the data are analysed in detail and the application of air-coupled impact-echo for the condition assessment of concrete sleepers is evaluated.

Specifications Table

**Table:** 

Subject	*Engineering & Materials science*
Specific subject area	*Non-destructive testing of concrete*
Type of data	*Tables and raw data of time series*
Data collection	*Air-coupled impact-echo (IE) actuation: air nozzle KN-R02–250 from “SMC Deutschland GmbH”* *electromechanical IE actuation: CTG-2 concrete thickness gauge-impact from “Olson Instruments Inc.”* *Manual IE actuation: IE hammer with an 8* *mm steel sphere* *IE sensing: Accelerometer model 352A60 from “PCB Piezotronics, Inc.”* *Analogue amplifier: 3-channel battery signal conditioner 480B21 from “PCB Piezotronics* *Inc.”* *The IE data is acquired as a voltage.* *Crack mapping: Visual inspection, Cracks are catalogued in respect to crack type and damage class according to* [1] *Data Acquisition: USB-6361 (National Instruments)* *Software: LabView (National Instruments) for data acquisition* *Photographs: Nikon Z6*
Data source location	*Institution: Bundesanstalt für Materialforschung und -prüfung - BAM (Federal Institute for Materials Research and Testing), Unter den Eichen 87, Test hall of Division 8.2* *City/Town/Region: Berlin* *Country: Germany*
Data accessibility	*Repository name: Zenodo* *Data identification number:*10.5281/zenodo.22144366 *Direct URL to data:*https://doi.org/10.5281/zenodo.22144366 *Instructions for accessing these data: A Matlab script to open, sort, evaluate, and visulise the measurement data are available online in a GitHub repository (URL:*https://github.com/ChristophStrangfeld/Air-coupled-impact-echo*) under a CC BY 4.0 licence*
Related research article	*C. Strangfeld, D. Ringeloth, Air-coupled Impact-echo Actuation Applied on Concrete Sleepers for Crack Detection. Journal of Nondestructive Evaluation 45, 116 (2026). DOI:*10.1007/s10921–026–01,404-z [2]

## 1 Value of the Data

• The first-ever published dataset of air-coupled impact-echo measurements.
• The first application of air-coupled impact-echo to a slender geometry, namely a type B70 prestressed reinforced concrete sleeper.
• The dataset provides the opportunity for a one-to-one comparison of different impact-echo actuators.
• The impact-echo dataset and the simultaneous crack mapping at every measurement position enable the investigation of correlations between signal features and component condition.
• The dataset can be used as a benchmark to develop new measurement and evaluation techniques and support the generation of guidelines and standards for non-destructive testing of concrete sleepers.

## 2 Background

In the laboratory, six prestressed concrete sleepers of type B70 were investigated. Sleepers 02 and 03 were factory-new and produced by the same manufacturer in the same batch in 2023. Sleepers 07 to 10 have been in service for several years in the railway system in the state of Thuringia, Germany. During track renewal, the sleepers were dismantled and stacked on top of each other next to the track and remained there for several years. For non-destructive testing, the sleepers were transported to BAM. All four sleepers show several severe cracks, especially around the sleeper heads.

The surface acceleration resulting from different IE actuations was measured. Measurements were performed in a 5 cm grid along the centre line of the sleeper top surface. Three different types of actuator were used. The spatial distance along the centre line between the accelerometer and the actuator was always 5 cm. The raw data are analogue time signals in volts recorded by the accelerometer. The cracks were catalogued in the same 5 cm spacing for the top and all lateral surfaces. The data were collected in an Excel file. The visual inspection of all sleepers was performed by the same person to maintain comparability between different sleepers.

## 3 Data Description

The dataset contains impact-echo data for three different types of actuators and tables of the crack mapping based on visual inspection. The impact-echo data were acquired by a calibrated accelerometer fixed to the concrete surface by means of an adhesive tape at a distance of 5 cm from the actuator. The actuators, accelerometer, and the measurement grid are shown in Fig. 1. All data are raw data as they were recorded in the laboratory. Measurements were recorded for a time period of two seconds with a sample rate of 250,000 S/s. At each sampling point, the measurement was repeated five times. To maximise comparability, all three actuators were investigated at every sampling point before the accelerometer position was changed.

**Fig. 1:**
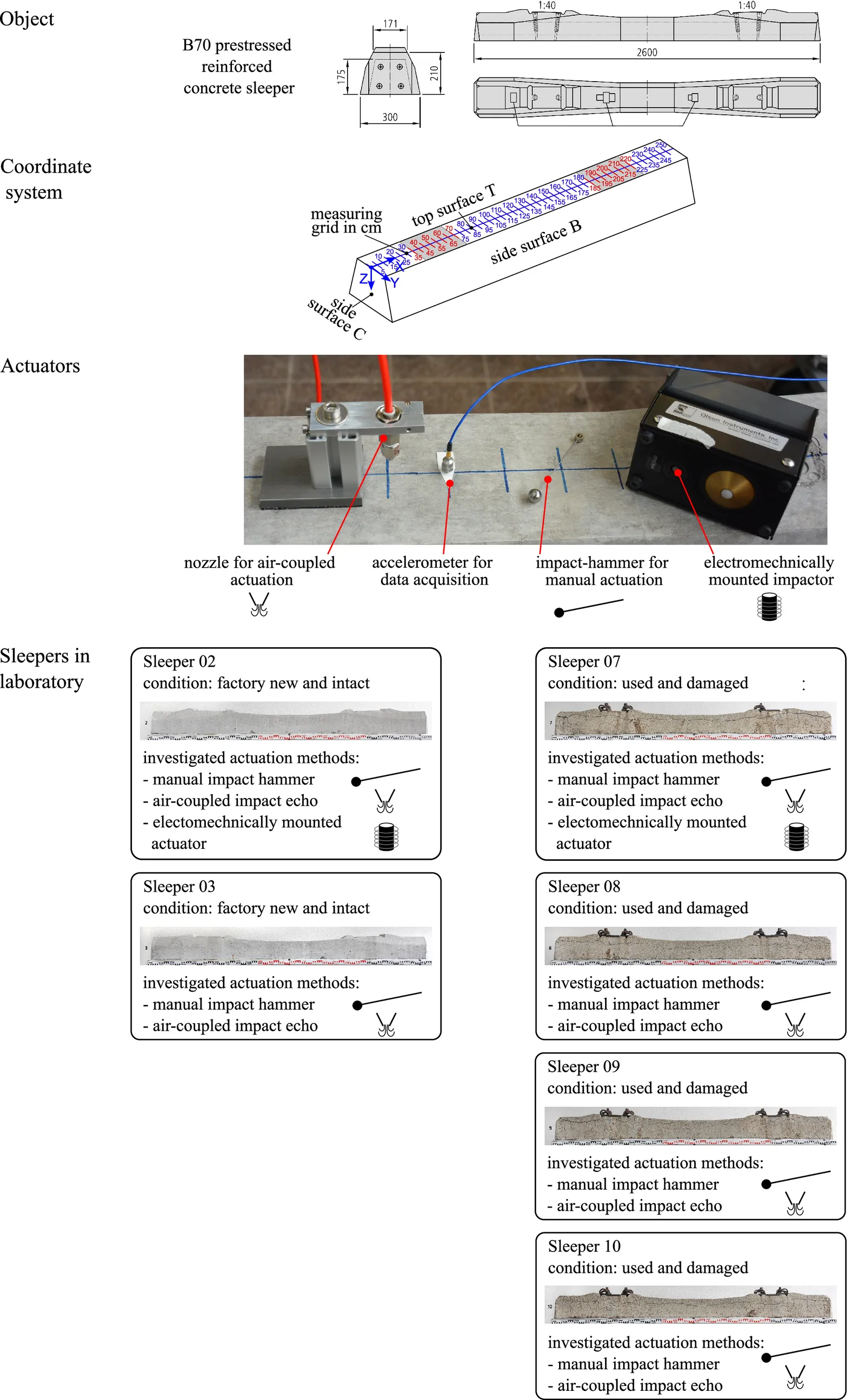
Overview of the design of experiments. Object: geometry and dimensions of type B70 sleepers; Coordinate system: origin, orientation, and sampling points on the top surface; Actuators: Photograph of the three different actuation types and the accelerometer used for data acquisition; Sleepers in laboratory: Photograph of side B of the six investigated sleepers and overview of the applied actuation methods.

Fig. 2 shows the time signal of the impact-echo signal measured on the intact sleeper 03 at X = 20 cm. The complete time signal is shown in Fig. 2a, including six clear peaks of the six manual impact-hammer actuations. Fig. 2b shows the same signal for a shorter time frame and a smaller voltage range. The signal amplitude decreases exponentially. For the air-coupled actuation, the complete time signal is shown in Fig. 2c. The input voltage range is significantly smaller. On the other hand, the actuation happens continuously, and thus no distinct peaks are visible. For comparison, Fig. 2d shows the signal for the same time frame as for the manual impact-hammer. Spectrograms and frequency analyses are available in [2].

**Fig. 2:**
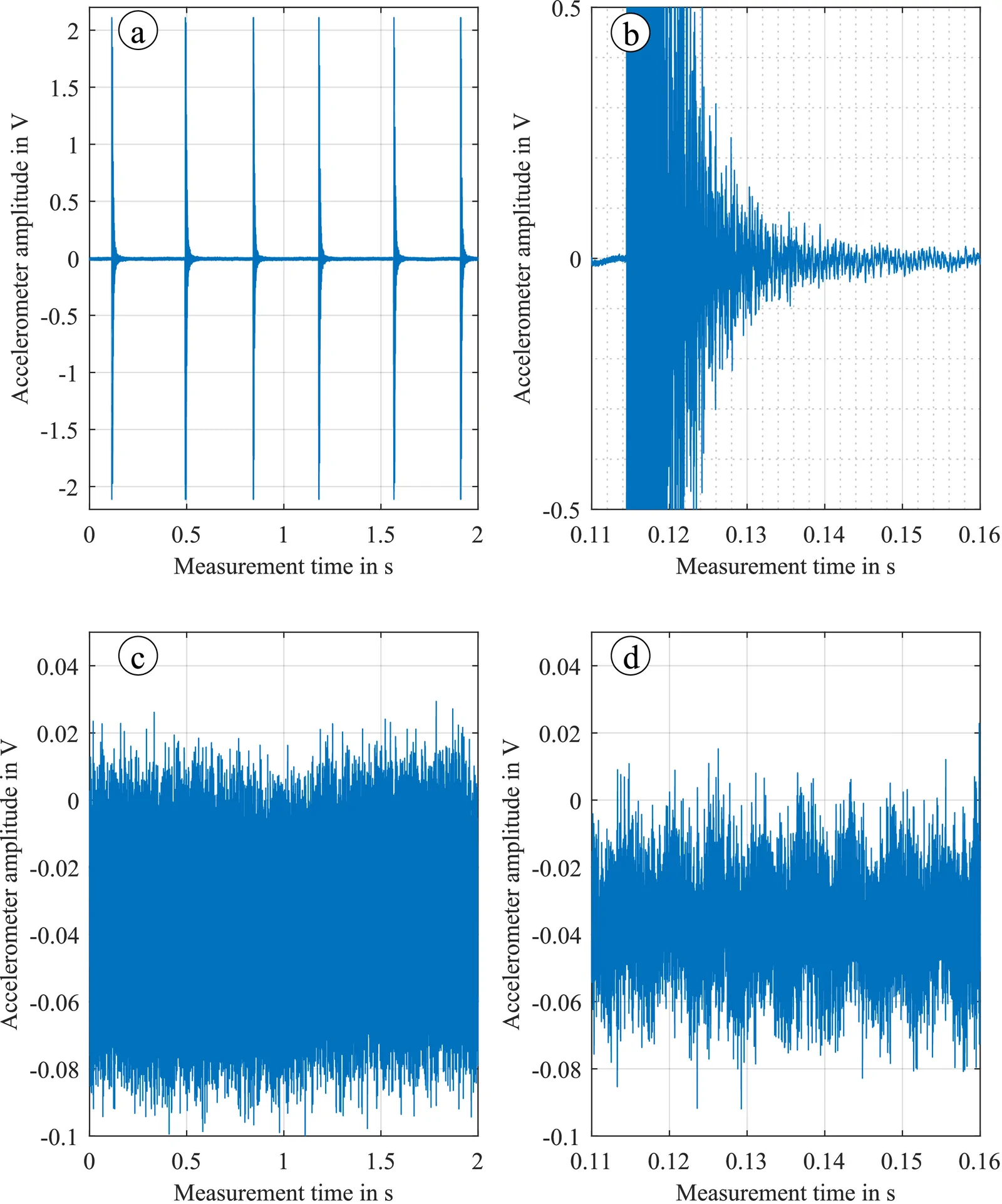
Time signal in V of the manual impact-hammer and the air-coupled actuation; a: Complete time signal including six manual impact-hammer actuations; b: Zoom of the time signal showing the exponential decay of the amplitude; c: Complete time signal of the continuous air-coupled actuation; d: Zoom of the time signal for the same time window as in subplot b.

The crack mapping of every damaged sleeper is catalogued in a single table. The factory-new sleepers were visually inspected as well. As no cracks were found in both intact sleepers, no crack catalogue was created. The catalogue is in accordance with the German guideline Ril-821 of 2018 [1]. Five damage classes (DC) are defined depending on the widths of the cracks. Thereby, damage class one (DC1) represents the most serious damage, such as gaping cracks, damage class four (DC4) stands for hairline cracks, and areas free of cracks have damage class five (DC5). Thus, the lowest damage class DC1 stands for the greatest damage, and the highest damage class DC5 symbolises the lowest damage, i.e., no damage. At each sampling point, starting at X = 0 cm, the damage class of the type of crack considered is documented.

The crack cataloguing was performed for each surface and crack type individually. The investigated crack types are longitudinal cracks, beading cracks, cross cracks, head cracks, and drift cracks. Longitudinal, beading, and cross cracks were catalogued on the top surface (T) and the two large side surfaces A and B. Head cracks were recorded only on the two small side surfaces C and D. Drift cracks were catalogued for all five surfaces. Thus, the first column in every table describes the crack type, the second column the investigated sleeper surface, and the third the damage class. If the damage class of the considered crack type on the investigated surface was observed at the current sampling point, it was documented with a value of 1. If no cracks were observed, DC5 was marked with 1 to indicate damage-free areas. At some sampling points, several cracks of the same crack type were visible at different depths. If these independent cracks had different damage classes, both damage classes were marked as observed. Therefore, at one sampling point for a specific crack type, more than one DC could be documented with one. If these cracks had the same damage class, this damage class was marked only once as observed. Not observed DC were marked with a zero in the table. In addition, for every crack type and surface, the minimum damage class is written as a sixth line below the five DC lines. This should give a quick overview, as the lowest damage class describes the highest damage. On the top surface, in the range of 40 cm ≤ X ≤ 65 cm and 190 cm ≤ X ≤ 215 cm, the rail mountings and damper pads are located and prohibit visual inspection and impact-echo measurements. Therefore, no damage class could be recorded in this range, and at these sampling points, the values in the table are marked as “-”. Fig. 3 depicts a section of the table for sleeper 07.

**Fig. 3:**
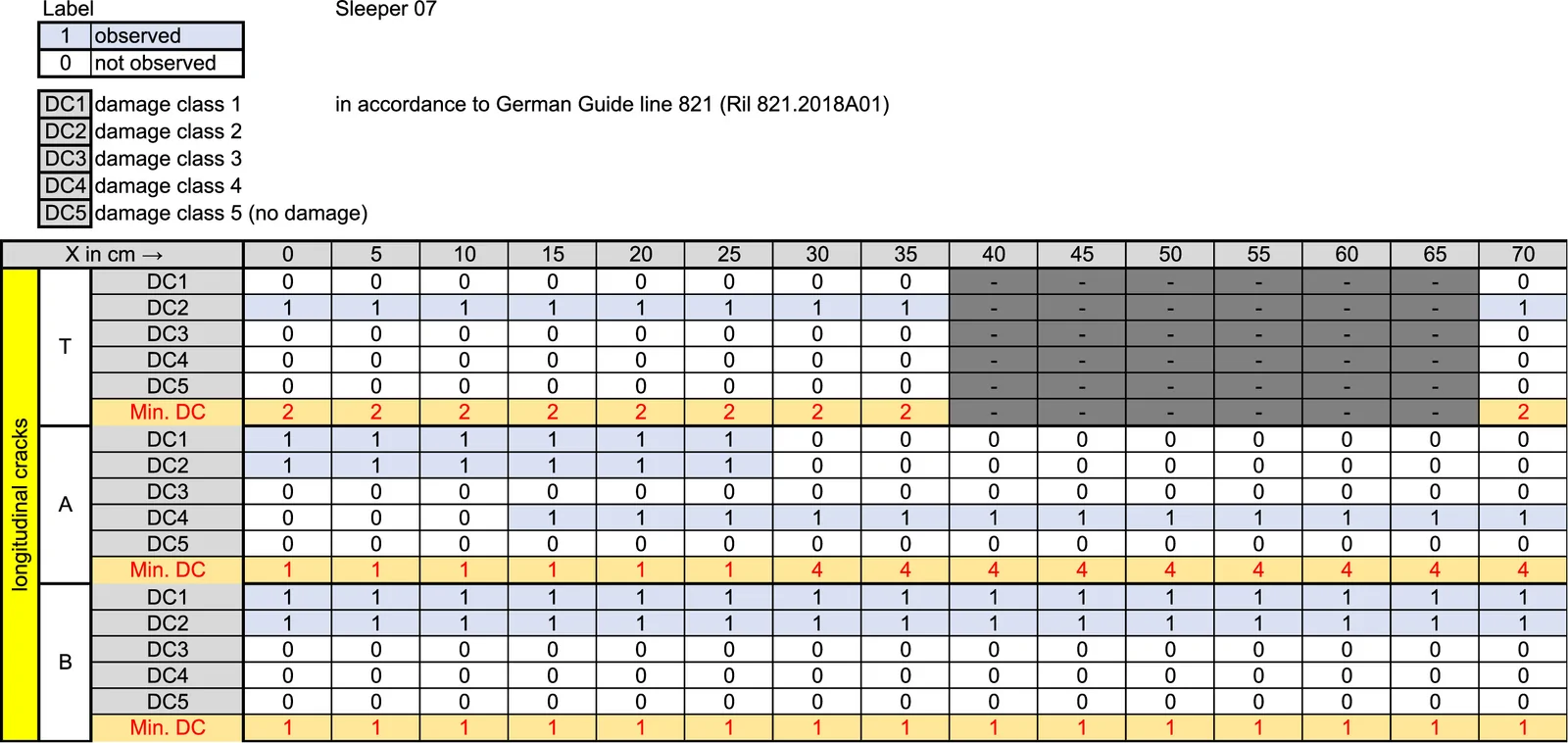
Section of the crack catalogue for sleeper 07. Damage classes for longitudinal cracks for the top surface T and the two side surfaces A and B are shown for the first 70 cm.

Fig. 4 shows the folder and file structures. The main folder contains six subfolders, each for one sleeper. There, the measurement data are stored in separate folders. The folder name describes the actuation method. Only for the four damaged sleepers 07 to 10, a crack catalogue is available. The electromechanical impact-echo actuation, abbreviated Olson in Table 2, was performed only on sleepers 02 and 07.

**Fig. 4:**
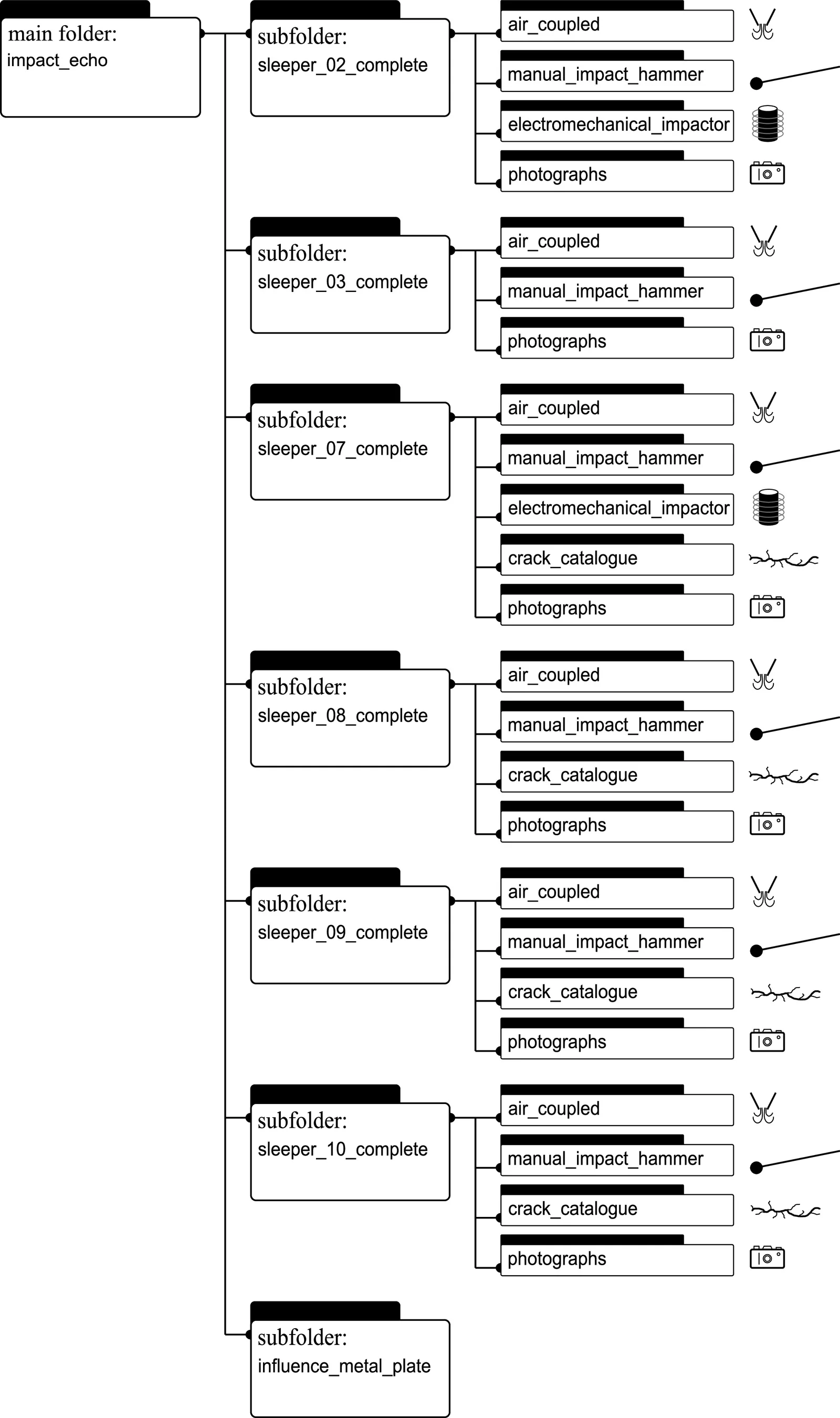
Overview of the folder and file structure.

Every actuation type is stored in a separate subfolder in the corresponding sleeper folder. Each subfolder contains 68 files, representing the 34 measurement positions available on the top surface of the sleeper. The file name always starts with the German name of the actuation type, followed by the number of the investigated sleeper, and the X-position along the centre line on the top surface. Table 1 shows the file name structure for every actuation type. Every table in the tdms-files contains seven columns and 500,000 lines. The first column is the measurement time in seconds, starting at zero. The next five columns are the analogue input voltage of the accelerometer for five repeated measurements. The last column is the average voltage value of the five measurements. In the current design of experiment, the average value delivers no reasonable value as the actuation has not been triggered and should not be considered.

**Table 1:** Actuation type and corresponding file name. The symbol # represents a digit.

Actuation type	File name
*air-coupled actuation*	*Luftgekoppelt SE## X=###.tdms*
*manual impact-hammer actuation*	*Handhammer SE## X=###.tdms*
*electromechanically mounted*	*Impaktor SE## X=###.tdms*

Every crack catalogue is saved in four different data formats, as a .xlsx Excel Worksheet file, a .pdf file, a UTF-8 .csv file, and a Unicode .txt file. These files are stored in a separate subfolder located in the folder of the considered sleeper. In this folder, there is a file named “Table_crack_mapping_sleeper_0X” that contains all crack types. Furthermore, there is a crack table for each individual crack type, i.e. longitudinal, beading, cross, head, and drift cracks. The header of every table is the same between lines 1 and 11. This should enable an automated reading of all tables.

## 4 Experimental Design, Materials and Methods

Six prestressed concrete sleepers of type B70 were investigated with impact-echo. To maximise comparability between the three actuators, the data recording was kept identical for all sleepers, actuators, and sampling points. The accelerometer and the actuators were always manually positioned. Impact-echo measurements were performed only on the top surface.

### 4.1 Materials

Fig. 5 shows the top and all side surfaces of the six investigated sleepers. Photographs were taken with a Nikon Z6 in the highest available quality. The aperture has been set manually to adjust for changing ambient daylight. For the four damaged sleepers, detailed photographs of the top surface and the two side surfaces A and B are available in the data repository. The six prestressed concrete sleepers of type B70 were investigated at the measurement points shown in Table 2. Sleepers 02 and 03 were produced by the same manufacturer in the same batch in 2023. They are factory-new and have never been used or subjected to any loads. Thus, they are considered intact. Minor concrete spalling occurred at the bottom edge during transport with a forklift. The four sleepers 07 to 10 were made available by the German centre for rail traffic research at the federal railway authority (DZSF), which is also the funding body of this research project.

**Fig. 5:**
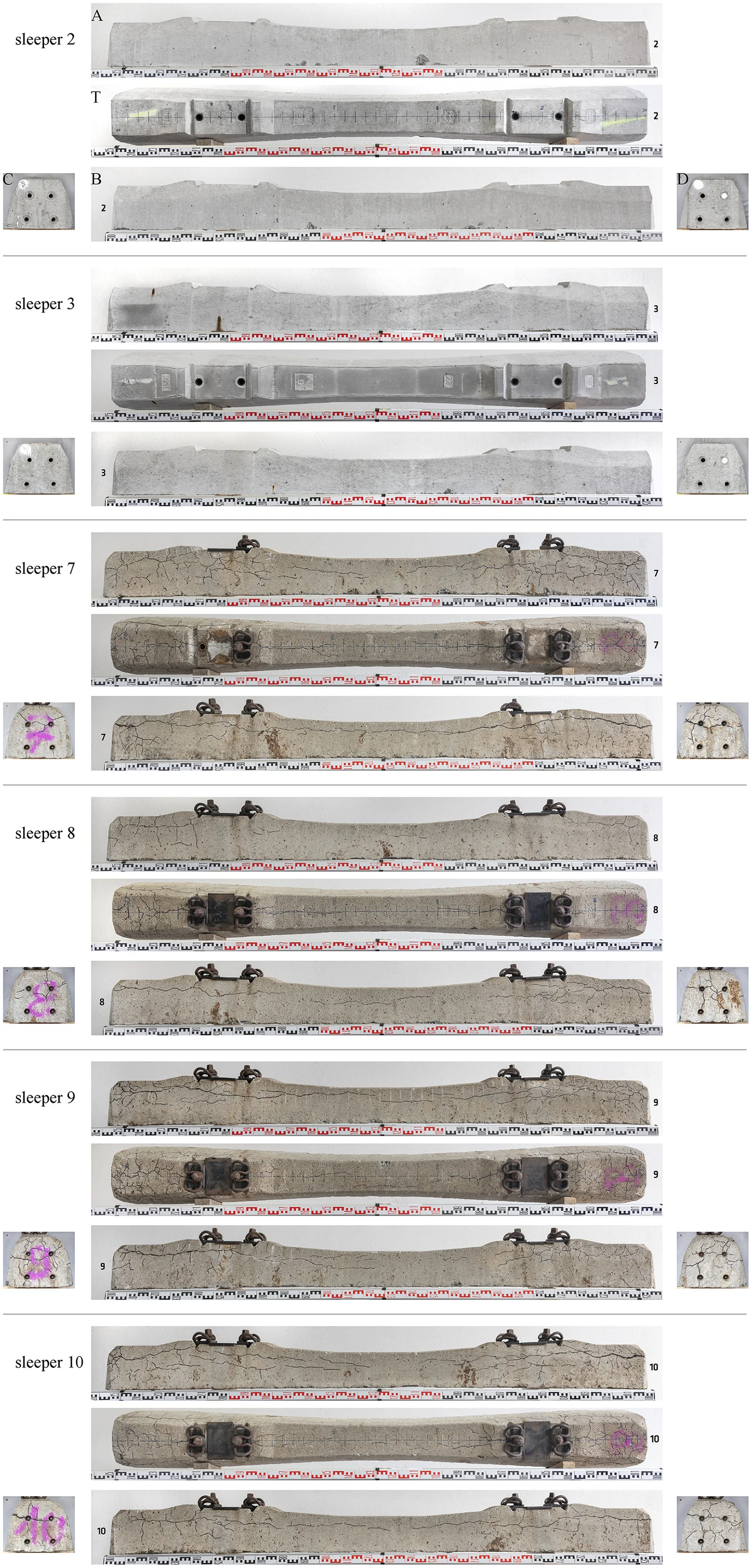
Overview of the six investigated sleepers.

**Table 2:** Overview of the investigated sleepers of the type B70.

Railway sleeper	Condition	X-position of year stamp	IE systems used
*sleeper 02*	*intact*	*X* *=* *95 cm*	*manual, air-coupled, Olson*
*sleeper 03*	*intact*	*X* *=* *95 cm*	*manual, air-coupled*
*sleeper 07*	*damaged*	*X* *=* *155 cm*	*manual, air-coupled, Olson*
*sleeper 08*	*damaged*	*X* *=* *155 cm*	*manual, air-coupled*
*sleeper 09*	*damaged*	*X* *=* *95 cm*	*manual, air-coupled*
*sleeper 10*	*damaged*	*X* *=* *95 cm*	*manual, air-coupled*

There is no documentation regarding these sleepers. They are known to have been in service for several years in the German railway system in the state of Thuringia, Germany. During routine track renewal, the sleepers were dismantled and stacked on top of each other, next to the track. They remained there for several years, continuously exposed to natural weather conditions. There is no information available regarding the type of track they were used in, the number of trains that passed over them, or the direction of traffic. The top surface of the sleepers is significantly deteriorated and eroded, so it is not even possible to read the engraved year of production anymore. When the sleepers arrived at Bundesanstalt für Materialforschung und -prüfung - BAM (Federal Institute for Material Research and Testing), they were first cleaned with a high-pressure water cleaner. The sleepers were stored in the test hall for more than three months before the first measurements were performed. The relative humidity in the hall remains below 50% throughout the year.

A measurement grid with a 5 cm spacing was drawn along the centre line of the top surface. On the bottom side, the sleeper is 260 cm long. All side surfaces are inclined towards the top surface. Thus, the top surface is around 256 cm to 257 cm long. To remain consistent with the measurement grid with a 5 cm spacing, the side surface D is assumed to be at 255 cm. X = 0 cm is located directly on the edge of side surface C, rendering measurements impossible. Thus, the first measurement was always taken at X = 5 cm. Although point X = 255 cm still has a distance of around 2 cm to the side edge, no measurements were taken. First, edge effects would probably distort the measurement signal significantly. Second, the number of measurement positions on each head should be identical to maintain symmetry in the dataset and subsequent plots. The measurement point X = 130 cm is shifted by around 2 cm to the geometric centre of the top surface, which is located at around X = 128 cm. Table 3 gives an overview of the 34 measurement points used.

**Table 3:** Overview of the measurement locations and their corresponding coordinates.

Position index	Measurement coordinate	Measurement region on sleeper
*# 1*	*X* *=* *5 cm*	*left head*
*# 2*	*X* *=* *10 cm*	*left head*
*# 3*	*X* *=* *15 cm*	*left head*
*# 4*	*X* *=* *20 cm*	*left head*
*# 5*	*X* *=* *25 cm*	*left head*
*# 6*	*X* *=* *30 cm*	*left head*
*# 7*	*X* *=* *75 cm*	*middle*
*# 8*	*X* *=* *80 cm*	*middle*
*# 9*	*X* *=* *85 cm*	*middle*
*# 10*	*X* *=* *90 cm*	*middle*
*# 11*	*X* *=* *95 cm*	*middle*
*# 12*	*X* *=* *100 cm*	*middle*
*# 13*	*X* *=* *105 cm*	*middle*
*# 14*	*X* *=* *110 cm*	*middle*
*# 15*	*X* *=* *115 cm*	*middle*
*# 16*	*X* *=* *120 cm*	*middle*
*# 17*	*X* *=* *125 cm*	*middle*
*# 18*	*X* *=* *130 cm*	*middle*
*# 19*	*X* *=* *135 cm*	*middle*
*# 20*	*X* *=* *140 cm*	*middle*
*# 21*	*X* *=* *145 cm*	*middle*
*# 22*	*X* *=* *150 cm*	*middle*
*# 23*	*X* *=* *155 cm*	*middle*
*# 24*	*X* *=* *160 cm*	*middle*
*# 25*	*X* *=* *165 cm*	*middle*
*# 26*	*X* *=* *170 cm*	*middle*
*# 27*	*X* *=* *175 cm*	*middle*
*# 28*	*X* *=* *180 cm*	*middle*
*# 29*	*X* *=* *225 cm*	*right head*
*# 30*	*X* *=* *230 cm*	*right head*
*# 31*	*X* *=* *235 cm*	*right head*
*# 32*	*X* *=* *240 cm*	*right head*
*# 33*	*X* *=* *245 cm*	*right head*
*# 34*	*X* *=* *250 cm*	*right head*

The sleepers had a random orientation when placed in the laboratory. Therefore, the X-position of the year stamp changes between X = 95 cm and X = 155 cm / either X = 95 cm or X = 155 cm. Since the sleepers are symmetrical, this is the only feature that can indicate their X-orientation, as shown in Table 2. The measurement grid of the intact sleeper S2 has partly disappeared in the photograph in Fig. 1. The adhesive tape used to fix the accelerometer did not hold at several positions. Therefore, the top surface of the sleeper has been slightly sanded. Then, the adhesive tape held well.

After the initial water-cleaning, the sleepers were always stored on wooden pallets in the test hall. For measurements, the investigated sleeper was positioned on two cuboidal steel support trestles. Their dimensions are 32 cm x 32 cm x 64 cm. Thus, the sleeper underside had a distance of 64 cm to the floor. During measurement, the 32 cm x 32 cm end surface was placed directly below the rail mountings at X = 35 cm to X = 70 cm and X = 185 cm to X = 220 cm, respectively. No mats, spacers, or blocks were placed between the sleeper and the support trestles. For the photographs only, a wooden block was placed between the sleeper and the trestles. The block was required to place the telescopic levelling staff aligned and parallel to the sleeper.

### 4.2 Visual inspection

After cleaning, all cracks were catalogued based on a visual inspection. All cracks were classified by the same person to ensure consistency and comparability. The top surface (in the X-Y-plane) and the two large side surfaces (in the X-Z-plane) and the two small side surfaces (in the Y-Z-plane) were investigated for the five crack types: longitudinal cracks, beading cracks, cross cracks, head cracks, and drift cracks. This was done at every sampling point. The damage class was determined by means of a crack width card and according to [1]. The card used started at 0.1 mm and increased in 0.05 mm steps to 1 mm. If cracks of the same type with different damage classes were observed at a single sampling point, all these damage classes were marked as observed. If cracks of the same type had the same damage class, this was marked only once, as observed in the tables. In the range of the rail mounts and the elastic pads, no cracks were documented on the top surface because they were not accessible. After all cracks were systematically catalogued, the cracks were traced with a black marker, regardless of their damage class. Therefore, the cracks of the photographed sleepers in Fig. 1, Fig. 5 appear much thicker than the real crack width.

For the damaged sleepers, detailed photographs of the top surface and the two side surfaces A and B are available. They can be used to determine the depths of the cracks. As a scale is added, the crack width and damage class (DC) can be estimated based on the digital images. Thus, more detailed crack catalogues of areas with multiple cracks can be generated.

### 4.3 Signal recording

The accelerometer was mounted using double-sided adhesive tape approximately 1 mm thick. The accelerometer used was model 352A60 and the analogue signal was amplified using the 3-channel battery signal conditioner 480B21, both manufactured by “PCB Piezotronics Inc.” [3]. The accelerometer was used to record the surface vibrations of all three actuators. In most cases, the actuator and accelerometer were positioned approximately 5 cm apart. The actuator was consistently positioned closer to the centre of the sleeper, resulting in the accelerometer being located about 5 cm further outward, closer to the corresponding sleeper head. The only exception occurred at the position closest to the outer rail mounting, where limited space required the accelerometer to be placed closer to the centre than the actuator. The measurement procedure was as follows: The accelerometer was fixed with adhesive tape. Then, measurements of all three actuation types were performed at these sampling points. After the signal recording of the three actuators was finished, the accelerometer was dismantled and fixed to the adjacent sampling point, including the use of new adhesive tape.

The accelerometer was calibrated by the company on the 23rd of August 2024, and its conversion was 10.43 mV/g or 1.063 mV/m/s² respectively. Based on the data sheet of the model 352A60, the root mean square value of the broadband resolution from 1 Hz to 10 kHz is 0.02 m/s² [3]. With a gain of 10 of the amplifier, the broadband resolution increases approximately to 0.2 mV rms. The analogue-to-digital converter of the 16-bit signal conditioner 480B21 is 0.027 mV rms based on the data sheet. The resolution of the analogue signal is 0.015 mV at an input range of ±0.5 V.

### 4.4 Actuators

Fig. 1 shows the three different actuation systems for impact-echo measurements on prestressed concrete sleepers.

#### 4.4.1 Air-coupled signal actuation

For air-coupled actuation, the male threaded air nozzle KN-R02–250 from “SMC Deutschland GmbH” was used. It is made of steel and is used for compressed air guns. The inner diameter is 2.5 mm, and the supply pressure is 2 bar. The distance between the exit of the nozzle and the concrete surface was set to 24 mm, comparable to the setup in [4]. The jet flow was orientated perpendicular to the surface. Due to the inclinations around the rail mountings, small deviations from the perpendicular orientation occurred in these ranges. During measurements, a 300 mm × 200 mm × 10 mm aluminium plate was placed between the nozzle and the accelerometer. The edge of the plate placed on the sleeper was covered with a foam rubber layer approximately 2 mm thick.

#### 4.4.2 Manual signal actuation

For manual actuation, a steel hammer was used. The handle had a diameter of 1 mm and the sphere on top had a diameter of 8 mm. The impact was performed by hand at an approximate repetition rate of two impacts per second.

#### 4.4.3 Electromechanical impact-echo actuation

The electromechanically mounted actuation was performed with the commercially available CTG-2 concrete thickness gauge-impact of the company “Olson Instruments Inc.”. The impactor is a metal cylinder of about 5 mm in diameter. It is 3.6 cm long, weighs 6.8 g heavy, and electromechanically mounted. An impact is triggered by pressing a button located on the device handle. The repetition rate was around one impact per second. Although the device includes an internal accelerometer, the signal was recorded using the externally affixed accelerometer to maintain consistency with the setup used for the other two actuators. Only sleepers 02 and 07 were investigated with this method.

### 4.5 Positioning tolerance

The accelerometer and the actuators were always positioned by hand. The coordinate system and the measurement grid are shown in Fig. 1. The accelerometer has a diameter across the corners of the hexagon of 10.7 mm. It was positioned on the adhesive tape, which covered the intersection of the two grid lines. Thus, the position accuracy of the accelerometer is estimated to be ±3 mm. The air-nozzle is aligned by eye by looking from above. This was possible as the nozzle and the supply tube are circular and centred. The centre was aligned to the grid lines. The position accuracy is estimated to be ±6 mm. The centre of the impactor of the electromechanical actuator is marked with two lines on the casing. Thus, the positioning is easy, and the estimated accuracy corresponds to the width of the grid lines of ±2 mm. With the manual impact-hammer, two to three times per second were achieved. At this repetition rate, a precise hit of the intersection of the grid lines is not possible anymore. Therefore, the accuracy is estimated to be ±15 mm.

### 4.6 Influence of the aluminium plate

During measurements with air-coupled actuation, an aluminium plate was placed between the sensor and the nozzle. Furthermore, the nozzle was positioned with a stand with a weight of around 1 kg. The weight was required to prevent the nozzle from lifting due to the momentum of the pressurised air. A parametric study was performed to quantify the effects of the devices. The different configurations with hammer or air-coupled actuation and without aluminium plate, with aluminium plate only, and with the nozzle stand only are summarised in Table 4. All measurements were performed on the intact sleeper 02. Measurements were recorded for a time period of two seconds with a sample rate of 250,000 S/s. At each sampling point, the recording was repeated five times.

**Table 4:** Overview of the measurement configurations.

Measurement number	001	002	003	004	005	006	007	008	009	010	011	012	013	014
*Sensor position in mm*	*100*	*100*	*100*	*100*	*100*	*100*	*100*	*100*	*100*	*100*	*100*	*100*	*100*	*100*
*Actuator*	*ham* *mer*	*ham* *mer*	*ham* *mer*	*ham* *mer*	*ham* *mer*	*ham* *mer*	*air*	*air*	*ham* *mer*	*ham* *mer*	*ham* *mer*	*air*	*air*	*air*
*Actuation position in mm*	*150*	*150*	*150*	*150*	*150*	*150*	*150*	*150*	*2500*	*2500*	*2500*	*2500*	*2500*	*2500*
*Plate position in mm*		*125*			*125*			*125*		*125*			*2475*	
*Stand position in mm*			*150*			*150*					*150*			*2500*

Fig. 6 shows the spectra with and without an aluminium plate at X = 12.5 cm while actuating with the air-nozzle with a supply pressure of 2 bar. The accelerometer was positioned at X = 10 cm and the air-nozzle at X = 15 cm with and without a plate between them.

**Fig. 6:**
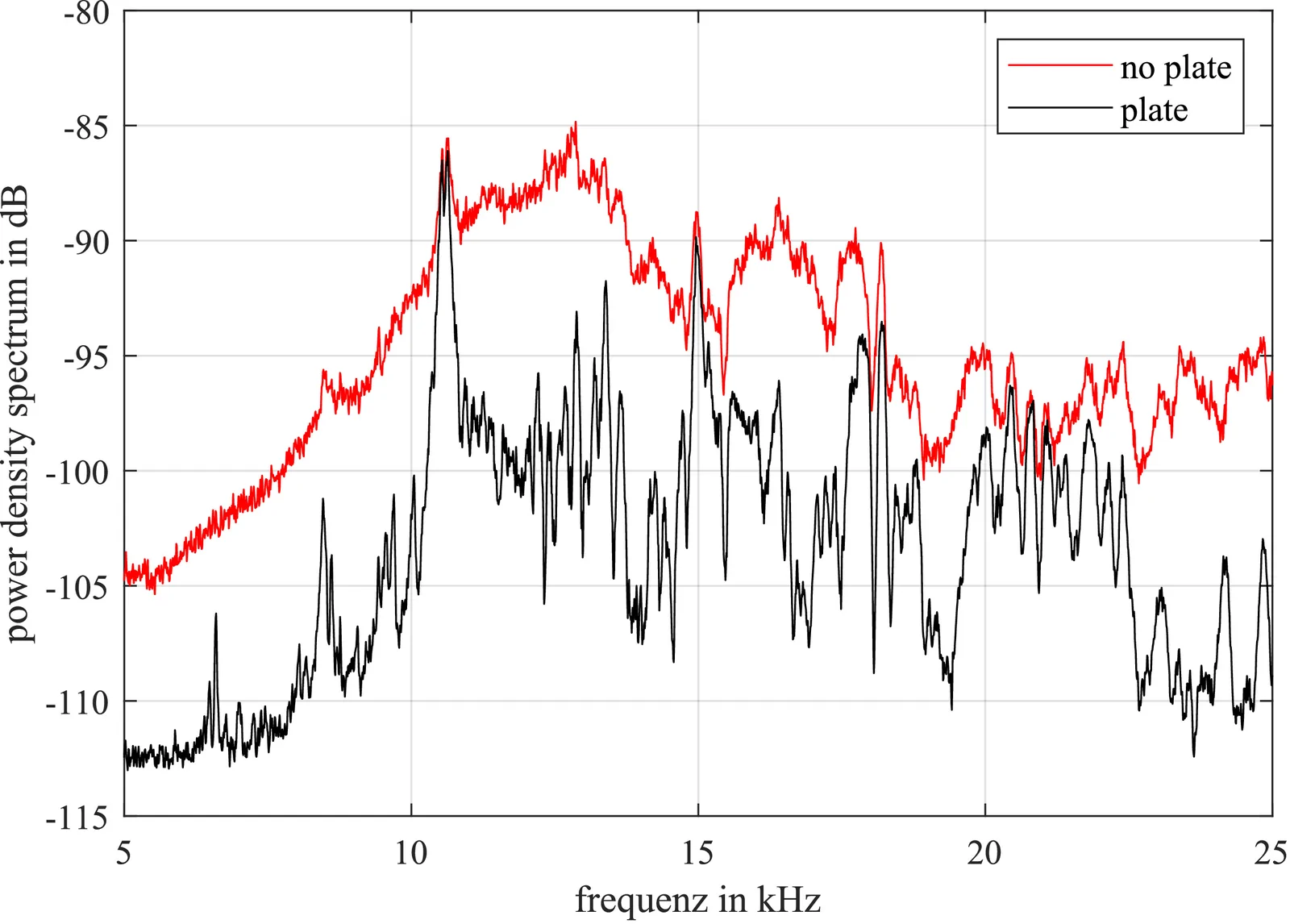
Comparison of the spectra obtained with the accelerometer at X = 10 cm and the air nozzle at X = 15 cm, with and without an aluminium plate placed between them.

Fig. 7 shows the spectra without and with the aluminium plate and nozzle stand. The actuation was done with the impact-hammer with a sphere diameter of 8 mm. The actuation was performed manually at X = 15 cm with respect to the positioning tolerance. The accelerometer was positioned at X = 10 cm, the plate at X = 12.5 cm, and the nozzle stand at X = 15 cm. During measurements with the nozzle stand and hammer actuation (without plate), the nozzle was switched off. Each of the three configurations was measured twice.

**Fig. 7:**
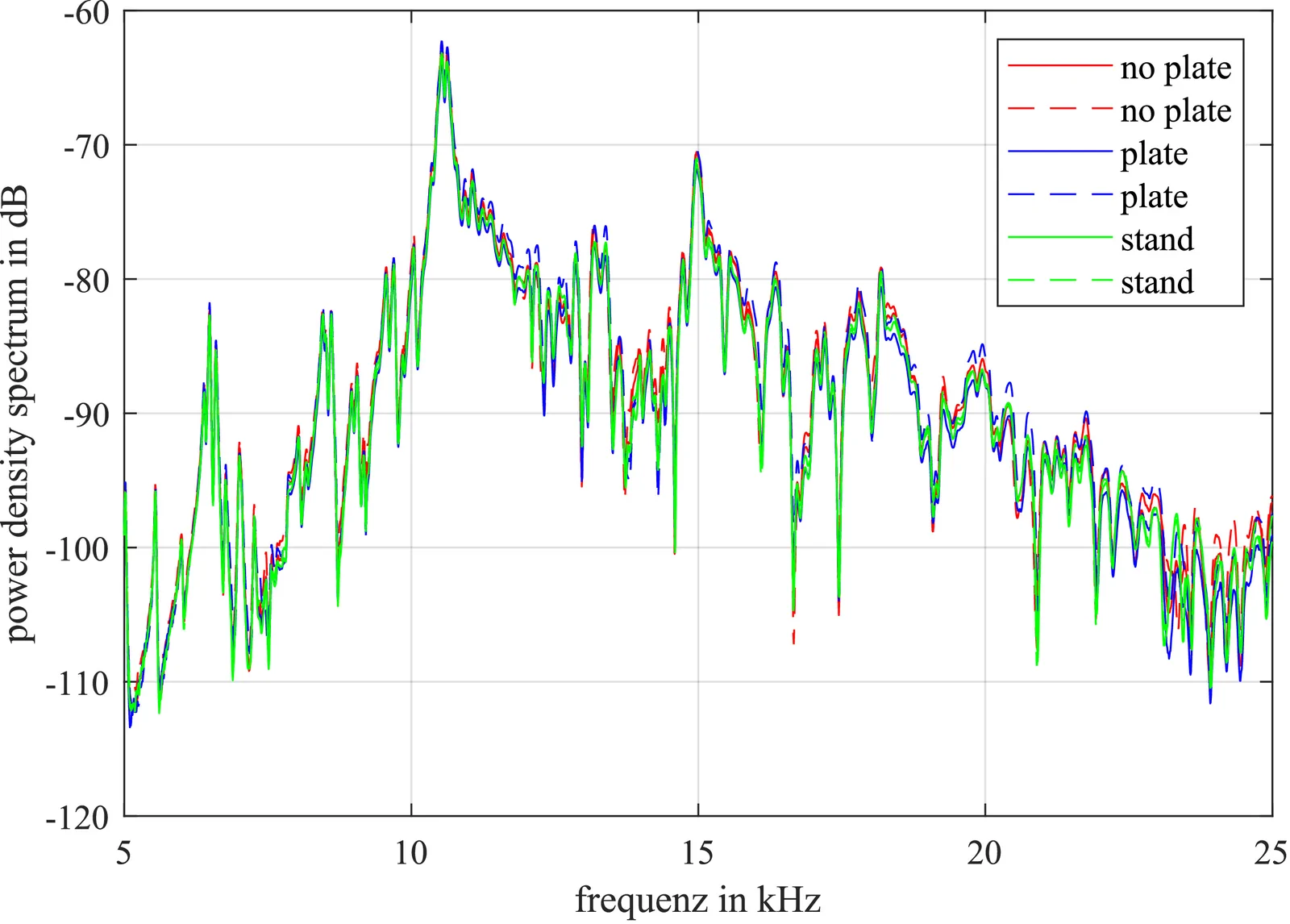
Comparison of the spectra with the accelerometer at X = 10 cm and impact-hammer actuation at X = 15 cm with and without an aluminium plate or nozzle stand.

Fig. 8 shows the spectra with a distance between the accelerometer and the actuator of 240 cm. The sensor was positioned at X = 10 cm, and the actuation at X = 250 cm. Measurements were performed without an aluminium plate, with the plate close to the accelerometer at X = 12.5 cm, and close to the actuation at X = 247.5 cm. The manual impact-hammer and the air-nozzle were both tested.

**Fig. 8:**
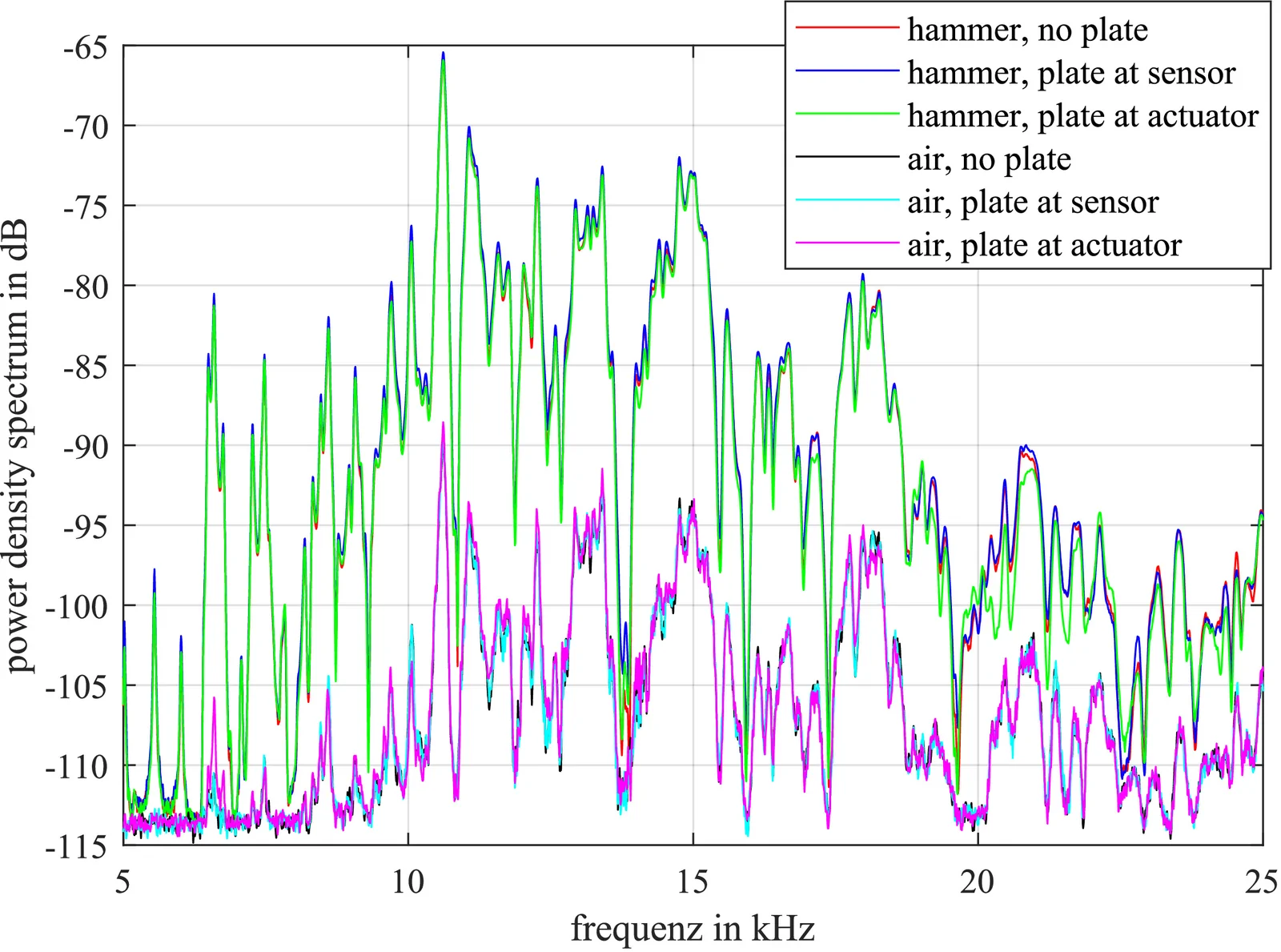
Comparison of the spectra with the accelerometer at X = 10 cm and impact-hammer and air-coupled actuation at X = 250 cm without aluminium plate or with the plate close to the sensors at X = 12.5 cm or close to the actuator X = 247.5 cm.

## 5 Limitations

The crack mapping and the catalogue are based on visual inspection. Thus, there is no information available on the depth, orientation, or merging of cracks inside the concrete structure or other internal defects.

## Ethics Statement

We confirm that all authors have read and follow the ethical requirements for publication in Data in Brief and the current work does not involve human subjects, animal experiments, or any data collected from social media platforms.

## CRediT Authorship Contribution Statement

The conception and creation of the entire text, as well as all figures and references, was done without the use of software based on artificial intelligence or large language models. All content was created by humans. Proofreading was performed by a professional human reader. Christoph Strangfeld: Funding, Resources, Conceptualisation, Methodology, Supervision, Project Administration, Data Curation, Software, Formal Analysis, Data visualisation, Writing - Original Draft, Writing - Review & Editing; David Ringeloth: Investigation, Data acquisition, Visual inspection, Crack cataloguing and characterisation, Writing - Review & Editing.

## Funding

This research project was funded by Deutsches Zentrum für Schienenverkehrsforschung beim Eisenbahn-Bundesamt (German Centre for Rail Traffic Research at the Federal Railway Authority) under the research grant 1113–111vb/012–0099#004, titled “Technische Machbarkeitsstudie zur Rissdetektion an Eisenbahn-Spannbetonschwellen mit Methoden der zerstörungsfreien Prüfung” (Technical feasibility study on crack detection in railway prestressed concrete sleepers using non-destructive testing methods). In project batch C (acoustic methods), BAM was a subcontractor of Siemens Mobility GmbH.

## Data Availability

(Zenodo).Impact-echo data of B70 prestressed reinforced concrete sleepers excited with three different actuators (Original data)
